# Traffic-Related Air Pollution and Otitis Media

**DOI:** 10.1289/ehp.9089

**Published:** 2006-04-26

**Authors:** Michael Brauer, Ulrike Gehring, Bert Brunekreef, Johan de Jongste, Jorrit Gerritsen, Maroeska Rovers, Heinz-Erich Wichmann, Alet Wijga, Joachim Heinrich

**Affiliations:** 1 University of British Columbia, School of Occupational and Environmental Hygiene, Vancouver, British Columbia, Canada; 2 GSF–National Research Center for Environment and Health, Institute of Epidemiology, Neuherberg, Germany; 3 Institute for Risk Assessment Sciences, Utrecht University, Utrecht, the Netherlands; 4 Department of Pediatrics, Division of Respiratory Medicine, Erasmus Medical Center–Sophia Children’s Hospital, Erasmus University, Rotterdam, the Netherlands; 5 Department of Pediatric Respiratory Medicine, University Medical Centre Groningen, University of Groningen, Groningen, the Netherlands; 6 Julius Centre for Health Sciences and Primary Care, University Medical Centre, Utrecht, the Netherlands; 7 Centre for Prevention and Health Services Research, National Institute for Public Health and the Environment (RIVM), Bilthoven, the Netherlands

**Keywords:** air pollution, cohort studies, infant, otitis media, vehicle emissions

## Abstract

**Background:**

Otitis media is one of the most common infections in young children. Although exposure to environmental tobacco smoke is a known risk factor associated with otitis media, little information is available regarding the potential association with air pollution.

**Objective:**

We set out to study the relationship between exposure to traffic-related air pollution and otitis media in two birth cohorts.

**Methods:**

Individual estimates of outdoor concentrations of traffic-related air pollutants—nitrogen dioxide, fine particles [particulate matter with aerodynamic diameters ≤ 2.5 μm (PM_2.5_)], and elemental carbon—were calculated for home addresses of approximately 3,700 and 650 infants from birth cohort studies in the Netherlands and Germany, respectively. Air pollution exposure was analyzed in relation to physician diagnosis of otitis media in the first 2 years of life.

**Results:**

Odds ratios (adjusted for known major risk factors) for otitis media indicated positive associations with traffic-related air pollutants. An increase in 3 μg/m^3^ PM_2.5_, 0.5 μg/m^3^ elemental carbon, and 10 μg/m3 NO_2_ was associated with odds ratios of 1.13 (95% confidence interval, 1.00–1.27), 1.10 (1.00–1.22), and 1.14 (1.03–1.27) in the Netherlands and 1.24 (0.84–1.83), 1.10 (0.86–1.41), and 1.14 (0.87–1.49) in Germany, respectively.

**Conclusions:**

These findings indicate an association between exposure to traffic-related air pollutants and the incidence of otitis media. Given the ubiquitous nature of air pollution exposure and the importance of otitis media to children’s health, these findings have significant public health implications.

Otitis media is one of the most common childhood infections in young children. Three of four children experience otitis media by 3 years of age, with most infections occurring before age 2 (Bluestone and Klein 2001). Otitis media is one of the leading causes of doctor’s visits in childhood (Freid et al. 1998) and the main reason for children to consume antibiotics or undergo surgery in developed countries (Rovers et al. 2004). Otitis media with effusion (OME), in which fluid and mucus stay trapped in the ear after infection, may lead to conductive hearing loss that, if persistent, may lead to delays in the development of speech, language, and cognitive abilities (Klein 2000; Teele et al. 1990). Recurrent acute otitis media leads to decreased quality of life measurements in children and is also stressful to their caregivers (Brouwer et al. 2005). In addition, the direct and indirect costs associated with otitis media are high: In the United States, annual health care costs were estimated at $3–5 billion (Bondy et al. 2000). Indirect costs due to caregiver work loss are also substantial and may in fact exceed direct costs. In 1994, the total yearly cost for otitis media in Canada was estimated to be $611 million—60% of the total economic cost associated with all forms of diabetes (Coyte et al. 1999). Evidence also indicates a steady increase in the incidence of diagnoses (Bluestone and Klein 2001; Lanphear et al. 1997). Consequently, identification of potentially preventable risk factors for otitis media, such as air pollution exposure, would have significant implications for health care costs. Because air pollution is not typically considered a risk factor for otitis media, this illness is also not considered in air pollution health impact and cost–benefit assessments (Kunzli et al. 2000).

Of particular relevance to a possible association between air pollution exposure and otitis media is the strength of environmental tobacco smoke (ETS) exposure as a risk factor (Jinot and Bayard 1996; National Cancer Institute 1999; U.S. Department of Health and Human Services 1986). A quantitative meta-analysis published in 1998 concluded that consistent evidence of causal relationship between parental smoking in the home and acute otitis media exists (Strachan and Cook 1998). Despite this finding and the large number of studies assessing the impact of ambient air pollution exposures on upper respiratory infections, the potential relationships between episodes of otitis media and ambient air pollution exposure have not been examined in detail. Accordingly, we assessed the relationship between traffic-related air pollution and otitis media in two birth cohorts, one in the Netherlands and another in Munich, Germany.

Previous analysis of this Dutch birth cohort indicated a significant association between a combined measure of severe upper respiratory tract and ear/nose/throat infections for the 12-month period before the child’s second birthday and exposure to traffic-related air pollutants (Brauer et al. 2002), but did not address otitis media specifically. This earlier analysis focused on the period between 12 and 24 months of age, whereas otitis media incidence peaks between 6 and 11 months of age (Rovers et al. 2004). Earlier analysis of the German cohort indicated associations between air pollution exposure and (nocturnal dry) cough without respiratory infections (Gehring et al. 2002). Neither of these analyses included independent assessment of otitis media episodes in relation to air pollution exposure.

## Materials and Methods

### Study populations

The Prevention and Incidence of Asthma and Mite Allergy (PIAMA) study is a prospective birth cohort study with an initial enrollment of 4,146 children (Brunekreef et al. 2002; Koopman et al. 2001; Wijga et al. 2001). The cohort was recruited in 1997–1998 during the second trimester of pregnancy from a series of communities varying from rural to large cities in the north, west, and center of the Netherlands. Mothers were classified as allergic or nonallergic on the basis of a validated screening questionnaire (Lakwijk et al. 1998). Nonallergic (based on a screening questionnaire) pregnant women were invited to participate in a “natural history” study arm (initial enrollment of 3,291). Pregnant women identified as allergic through a screening questionnaire were allocated primarily to an intervention arm (initial enrollment of 855), with a random subset allocated to the natural history arm. The intervention involved the use of mite-impermeable mattress and pillow covers. The study protocol was approved by the institutional review boards of each participating institute, and parents or guardians of all subjects gave written informed consent. All subjects with completed questionnaires at 2 years of age were included in the analyses.

The participants of the LISA-Munich (Influence of Lifestyle factors on the development of the Immune system and Allergies in East and West Germany) birth cohort study were recruited during pregnancy. From December 1997 to January 1999, newborns from six obstetrical clinics in Munich whose parents were born in Germany and had German nationality were defined as the target population for the study. A detailed description of selection, exclusion criteria, and characteristics of study population has been published previously (Gehring et al. 2002). For this analyses, we selected all infants with birth addresses in Munich (without surrounding communities) for whom questionnaire data were available for the first year of life and who did not move away from Munich within the first year of life. A total of 673 subjects from the LISA cohort fulfilled these criteria. The study was approved by the ethics commission of the Landesaerztekammer Bavaria and was carried out in accordance with the institutional guidelines for the protection of human subjects. Parents or guardians of all subjects gave written informed consent.

### Exposure assessment

Air pollution concentrations at the home address of each member of the cohort were calculated by combining air pollution measurements with a geographic information system (GIS; Brauer et al. 2003; Gehring et al. 2002). Briefly, air pollutants were measured at 40 individual sites in each country, designed to capture the maximum variability in pollution from traffic sources. Fine particles [particulate matter with aerodynamic diameter ≤ 2.5 μm (PM_2.5_)] were collected with Harvard impactors (Air Diagnostics and Engineering, Harrison, ME, USA), nitrogen dioxide was collected by Palmes tubes, and light-absorbing carbon was measured as the reflectance of the PM_2.5_ filters, a method shown to be highly correlated with thermal elemental carbon measurement (Cyrys et al. 2003). At each location, measurements were conducted for four 2-week periods dispersed throughout 1 year and then adjusted for temporal trends, based on the difference between concentrations measured during each period to annual average measurements (Hoek et al. 2002), to calculate long-term average concentrations for the 40 locations.

Geographic data were also collected regarding traffic, road, and population density in the vicinity of each monitoring location. We developed regression models to relate the annual average concentrations at the 40 monitoring sites with the geographic variables. For example, in the Netherlands, the number of high-traffic roads within a 250-m radius of a location, the presence of a major road within a 50-m distance, the density of buildings (addresses) within a 300-m radius, and an indicator for the region of the country were used in the model to predict light-absorbing carbon concentrations. In Munich, the light-absorbing carbon model used traffic intensity within a 50-m distance, traffic intensity in a circular area between 50 and 250 m, and the population density within a 300-m radius and in a circular area between 300 and 5,000 m to predict concentrations. Models with similar variables describing traffic intensity were developed for PM_2.5_ and NO_2_. These models explained 73, 81, and 85% of the variability in the annual average concentrations for PM_2.5_, light-absorbing carbon, and NO_2_, respectively, in the Netherlands (Brauer et al. 2003) and 56, 67, and 62% of the variability in the annual average concentrations for PM_2.5_, light-absorbing carbon, and NO_2_, respectively, in Germany (Gehring et al. 2002). We then applied these models to the same geographic variables measured at the home addresses of each individual in the cohort to obtain unique long-term ambient air pollution concentrations at the home address of each cohort member at the time of birth. Given our interest in early-life exposures, we estimated air pollutant concentrations only for the birth addresses of cohort members. However, in both locations only a small (9% in the Netherlands, 11% in Munch) percentage of the study population moved within the first year of life (Brauer et al. 2002; Gehring et al. 2002).

### Questionnaire data

We assessed information on otitis media using a parent-completed questionnaire. Specifically, in the Netherlands the question “Did a doctor diagnose infection of the middle ear in your child in the last 12 months?” was asked when the child was 12 months and 24 months of age. In Munich, the question “Did a doctor diagnose otitis media in your child during the last 6 months?” was asked every 6 months. A series of potential confounding variables (listed in Table 1) were selected if exploratory data analysis suggested substantial variability within the cohort or if variables were suspected of being risk factors for otitis media. Confounder data were selected from the earliest questionnaire that was available, to coincide with the exposure data that were estimated for addresses at birth. ETS exposure was assessed by questionnaire (Table 1) and has been validated against home nicotine measurements for the Dutch cohort (Brunekreef et al. 2000).

### Statistical analysis

We performed multiple logistic regression analyses to analyze the relationship between otitis media and estimated air pollution exposure for study subjects in each of the cohorts. Results are presented as crude and adjusted odds ratios (ORs) with 95% confidence intervals (CIs). We adjusted for potential confounding factors such as sex, parental atopy, maternal education, siblings, maternal smoking during pregnancy, ETS exposure at home, use of gas for cooking, indoor moulds and dampness, number of siblings, breast-feeding, and presence of pets in the home (Table 1). ORs are presented for standardized (between the two study locations) differences (approximating interquartile range differences in the two locations) in estimated exposures of 3 μg/m^3^ for PM_2.5_, 0.5 × 10^−5^/m for particle light absorbance [corresponding to ~ 0.5 μg/m^3^ elemental carbon, based on colocated sampling (Cyrys et al. 2003)], and 10 μg/m^3^ for NO_2_.

## Results

In both cohorts, the prevalence of otitis media increased in the second year and was nearly identical at 2 years of age (Table 1). By that age, approximately 35% of each cohort had at least one occurrence of otitis media. The cohorts were similar with respect to maternal age and parental allergy/atopy. The rate of breast-feeding was much higher in the German cohort, whereas ETS exposure, the use of gas for cooking, and the presence of pets were much higher in the Dutch cohort. The German cohort had a somewhat higher level of education and a lower number of siblings.

Exposures to traffic-related air pollution are summarized in Table 2. Median and mean concentrations of light-absorbing carbon and NO_2_ were similar between the Netherlands and Germany whereas PM_2.5_ concentrations were higher in the Netherlands, probably due to higher regional background concentrations. Interquartile ranges were nearly twice as large in the Netherlands (3.2 μg/m^3^, 0.54 10^−5^/m, and 16.4 μg/m^3^ for PM_2.5_, light-absorbing carbon, and NO_2_, respectively) than in Munich (1.5 μg/m^3^, 0.34 10^−5^/m, and 8.5 μg/m^3^), reflecting the fact that the Dutch cohort included suburban and semirural areas that were not heavily affected by traffic-related air pollutants, whereas the German cohort was restricted to the Munich metropolitan area in which traffic emissions contribute to the urban background as well as to variability within the area.

Crude ORs indicated elevated risks for otitis media in association with all air pollutants, with associations reaching statistical significance in the larger Dutch cohort (Table 3). These ORs were similar for otitis media in the first year of life and for cumulative incidence over the first 2 years. ORs increased slightly but were largely unaffected by adjusting for covariates in both cohorts. Given the differences in prevalence of covariates between the two cohorts, this suggests sufficient control for potential confounding variables or that these covariates did not have a major impact in this analysis. Further, we specifically investigated the impact of ETS exposure in both cohorts but did not observe any association between ETS exposure or smoking during pregnancy and otitis media, and adjustment for these exposures exposure did not change the associations between air pollution and otitis media.

Because attendance at a child care facility has been independently associated with respiratory tract infections in the Dutch cohort (Koopman et al. 2001) and is also a risk factor for otitis media occurrence (Bluestone and Klein 2001), we conducted a sensitivity analysis to evaluate its potential impact on the association with air pollution. Because child care attendance in Munich was very low—3% in the first year and < 15% at 2 years of age—and to retain similar analyses in both cohorts, we did not include child care attendance in the primary models. We therefore restricted the sensitivity analysis to the Dutch cohort that had higher levels of child care attendance. Of the 981 children (26%) who reported attending child care at 1 year of age, the median number of hours of child care attendance per week was 18, and 735 of these children attended child care > 10 hours per week. By 2 years of age, 1,256 children (34%) had reported attending child care. Adjusted ORs for otitis media with models incorporating child care attendance were somewhat reduced [e.g., for NO_2_ OR = 1.13 (95% CI, 0.99–1.28) at 1 year and OR = 1.10 (95% CI, 0.99–1.23) after 2 years of age] but still elevated. Additional stratified analyses indicate that the effect of air pollution on otitis media occurrence was not restricted to those children who attended child care.

## Discussion

This analysis represents the first examination of the relationship between air pollution exposure and otitis media in a large cohort study. In two birth cohorts with a common exposure assessment approach, we have identified associations between individual estimates of traffic-related air pollution exposure and the incidence of otitis media. Given the widespread nature of air pollution exposure and the high prevalence of otitis media, these findings indicate an important and previously unrecognized societal impact of air pollution. Further, given the high direct and indirect costs associated with otitis media episodes, our results suggest a potentially important preventable risk factor for this common childhood disease.

In contrast to limited previous analyses that have been conducted with small study populations and have focused largely on cross-sectional comparisons between different geographic regions (Heinrich and Raghuyamshi 2004), we assessed exposure at the individual level with individual control for covariates. Perhaps the strongest prior evidence relating air pollution with otitis media comes from one of our earlier studies in which prevalence rates for otitis media among 7,000 school-age children in three East German areas (two polluted and one control area) were compared in repeated cross-sectional surveys (Heinrich et al. 2002). In addition, temporal changes of prevalence rates for otitis were studied in parallel with dramatic improvements in air quality [sulfur dioxide and total suspended particles (TSP)] after German reunification (Heinrich et al. 2000). Although adjusted lifetime prevalence rates for otitis did not differ among children who grew up in areas with different levels of these air pollutants (Heinrich et al. 2002), there were significant increases in prevalence of other nonallergic respiratory illnesses (bronchitis, frequent colds, sinusitis, cough) and decreased lung function in children from the polluted areas (Frye et al. 2003; Heinrich 2003; Heinrich et al. 2002). In parallel with the decreases in SO_2_ and TSP in all three areas, prevalence rates for otitis decreased from 31% in 1992–1993 to 26% in 1995–1996 and 27% in 1998–1999 (Heinrich et al. 2002). The adjusted OR for a 50-μg/m^3^ change in TSP was 1.45 (95% CI, 0.89–2.37) and 1.42 (95% CI, 0.94–2.15) for a 100-μg/m^3^ change in SO_2_ concentration (Heinrich et al. 2002). However, these increased risk estimates of ambient air pollutants for otitis media were driven mainly by the temporal improvement of air quality and therefore may also parallel other unmeasured lifestyle changes, whereas the regional gradient of air pollution concentrations were not consistent with area-specific differences in prevalence rates. For several other nonallergic respiratory outcomes, temporal changes of prevalence rate (decreasing) were consistent with spatial differences (highest prevalence rate in the most polluted area).

In a somewhat similar design, ear infections were examined in two cross-sectional surveys of approximately 400 children 11–13 years of age from three districts of São Paulo, Brazil, conducted in 1986 and in 1998 (Ribeiro and Cardoso 2003). The three districts experienced different levels of SO_2_ in 1973–1983, and these differences were associated with crude prevalence rates (unadjusted for potential confounders) for current and frequent ear infection. Social indicators such as parental education and literacy also differed between the three districts. Further, temporal changes in ambient particulate matter concentrations were also associated with changes in frequent ear infection prevalence. Although this study has several limitations (small sample sizes, descriptive presentations of methods and main findings), the results are consistent with an association between prevalence of otitis media and ambient air pollution.

Other studies that have evaluated air pollution and otitis media typically lack exposure measurements or were conducted on small sample sizes (Caceres Udina et al. 2004; da Costa et al. 2004; Dostal et al. 2001; Holtby et al. 1997) but also suggest associations. For example, a study of 1,156 children with OME in the United Kingdom did not measure exposures but used distances of the home of these children from known industrial emission points as an exposure proxy (Holtby et al. 1997). A significantly greater portion of study entrants with OME lived within a 1,000-m buffer of an industrial point source, but no trend of decreasing prevalence rate of OME with increasing distance was observed.

Although our study has several major advantages over previous investigations (individual level exposure assessment and large study population), there are also inherent limitations to our approach. First, as is common with air pollution epidemiologic analyses, we estimated exposures instead of directly measuring them using personal monitoring. Additionally, for those children who moved or attended child care, estimating exposures at these locations may reduce exposure misclassification. Second, we asssessed otitis media by questionnaire-based self-reporting of physician diagnosis and not by any objective measure. Third, we did not assess severity or address issues such as recurrent otitis media and interactions between air pollution and different treatment regimes. Such analyses, although important to understanding the public health significance of our findings, are probably best addressed in a longitudinal study design in which detailed information regarding otitis media occurrence and treatment is evaluated in relation to short-term changes in air pollution exposure. Finally, although we adjusted analyses for a large number of potential risk factors, the possibility for residual confounding remains, especially given that exposure estimates were based on spatial contrasts in air pollution that may also lead to spatial contrasts for other unmeasured otitis media risk factors. These limitations are, however, largely unavoidable for cohort studies of large populations.

These findings indicate an association between exposure to traffic-related air pollutants and the incidence of otitis media. This association is supported by a wealth of evidence linking exposure to high levels of air pollution indoors in developing countries with acute lower respiratory infections (Smith et al. 2000), more limited evidence of associations between levels outdoor air pollution in developed countries and upper respiratory tract infections (Chauhan and Johnston 2003; Lin et al. 2005; Romieu et al. 2002), and the fact that some upper respiratory tract infections may progress to otitis media (Rovers et al. 2004). The strong evidence linking otitis media with ETS exposure and the similarities between ETS and ambient air pollution add further support to our findings. The specific air pollutants that affect respiratory infections have not been clearly identified, although some evidence suggests that NO_2_ and coarse particles may be especially active in this regard (Chauhan and Johnston 2003; Lin et al. 2005). Additionally, the mechanism by which air pollution may lead to otitis media is not known. Air pollution exposure may result in a more severe or persistent infection—for example, by decreasing mucociliary clearance (Chauhan and Johnston 2003; Thomas and Zelikoff 1999)—making progression to otitis media more likely. For example, an interaction between respiratory syncytial virus infection and NO_2_ exposure before infection has been demonstrated to lead to increased severity of asthma exacerbations (Chauhan et al. 2003). Alternatively, air pollution may actively promote progression to otitis media. Addressing these or other possibilities will require further research. Although replication of our results in similar cohort studies is needed, the ubiquitous nature of air pollution exposure and the importance of otitis media to children’s health suggest that these findings have significant public health implications.

## Figures and Tables

**Table 1:** Prevalence of otitis media and selected potential confounders in the two cohorts.

	The Netherlands		Munich, Germany	
Variable (timing of questionnaire)	*n*/*N*	(%)	*n*/*N*	(%)
Otitis media^a^				
At 1 year of age	667/3,714	18.0	108/665	16.2
At 2 years of age (cumulative)	1,262/3,650	34.6	226/650	34.8
Confounding variables^b^				
Female sex	1,899/3,934	48.3	328/673	48.7
Parental atopy			420/662	63.4
Maternal allergy	1,281/4,114	31.1		
Paternal allergy	1,172/4,051	28.9		
Mother smoking during pregnancy	583/4,079	14.3	100/646	15.5
Breast-feeding (first 3 months exclusively)			472/669	70.6
Breast-feeding (any at 3 months of age)	1,827/3,883	47.0		
ETS at home (3 months or 6 months of age)^c^	1,121/3,903	28.7	97/669	14.5
Maternal age at birth [median years (range)]	30 (17–42)		33 (19–44)	
Maternal education				
< 12 grades	1,743/3,709	47.0	219/670	32.7
≥ 12 grades	1,966/3,709	53.0	451/670	67.3
Siblings (at time of birth)	1,986/3,919	50.7	277/673	41.2
Child care attendance				
At 1 year of age	981/3,734	26.3	22/667	3.3
At 2 years of age (cumulative)	1,256/3,723	33.7	90/658	13.7
Use of gas for cooking (at 3 months of age)	3,236/3,911	82.7	89/672	13.2
Home dampness (at 3 months of age)			35/672	5.2
Indoor molds (at 3 months of age)	1,215/3,704	32.8	209/673	31.1
Pets (at 3 months of age)	2,011/3,905	51.5	118/672	17.6
Cat	1,283/3,905	32.8	53/671	7.9
Dog	629/3,905	16.1	23/671	3.4

*N* refers to the total number of subjects who provided information on the specific variable; *n* refers to the number of subjects answering affirmatively with respect to each variable.

a Otitis media responses refer to specific questions as described in “Materials and Methods.”

b Atopy (parental history of asthma and/or hay fever and/or eczema) was self-reported in the Munich cohort, and in the Netherlands atopy was self-reported allergy or reporting of physician-diagnosed allergy to house dust, house dust mite, pets, or hay fever/rhinitis) in the (expecting) mothers. In the Netherlands, indoor molds refers to the (self-reported) presence of molds, water damage, and visible moisture in any of four specified rooms. In Munich, indoor molds refers to self-reported molds or mildew or moisture spots anywhere in the dwelling.

c Question regarding exposure to ETS at home (“Does anyone smoke in your house?”) was asked at 3 months of age in the Netherlands and at 6 months of age in Munich.

**Table 2:** Distribution of estimated annual average air pollution concentrations for the home (birth) address in the cohorts.

	The Netherlands			Munich, Germany		
	PM_2.5_ (μg/m^3^)	Light-absorbing carbon (PM_2.5_ absorbance, 10^−5^/m)	NO_2_ (μg/m^3^)	PM_2.5_ (μg/m^3^)	Light-absorbing carbon (PM_2.5_ absorbance, 10^−5^/m)	NO_2_ (μg/m^3^)
Minimum	13.5	0.77	12.6	12.0	1.40	19.6
10th percentile	14.0	1.16	14.8	12.2	1.47	21.7
25th percentile	15.0	1.38	18.9	12.5	1.54	22.9
50th percentile	17.3	1.78	26.1	13.0	1.70	26.5
Mean	16.9	1.72	25.6	13.4	1.76	27.7
75th percentile	18.2	1.92	29.2	14.0	1.88	31.4
90th percentile	19.1	2.19	35.3	14.8	2.10	34.8
Maximum	25.2	3.68	58.4	21.9	4.39	64.4

**Table 3:** Association between long-term exposure to air pollution and otitis and respiratory infections in the two cohorts: crude and adjusted ORs and 95% CIs.

	The Netherlands				Munich, Germany			
	Unadjusted		Adjusted^a^		Unadjusted		Adjusted^a^	
Otitis media	OR (95% CI)	*N*	OR (95% CI)	*N*	OR (95% CI)	*N*	OR (95% CI)	*N*
At 1 year of age								
PM_2.5_	1.13 (1.00–1.29)*	3,705	1.13 (0.98–1.32)	2,984	1.09 (0.68–1.75)	665	1.19 (0.73–1.92)	620
Light-absorbing carbon	1.11 (1.00–1.23)*	3,705	1.11 (0.98–1.26)	2,984	1.07 (0.80–1.44)	665	1.12 (0.83–1.51)	620
NO_2_	1.14 (1.02–1.27)*	3,705	1.17 (1.03–1.34)*	2,984	1.03 (0.74–1.43)	665	1.09 (0.78–1.54)	620
At 2 years of age (cumulative)								
PM_2.5_	1.10 (0.99–1.22)	3,642	1.13 (1.00–1.27)*	2,970	1.18 (0.81–1.75)	650	1.24 (0.84–1.83)	605
Light-absorbing carbon	1.08 (0.99–1.18)	3,642	1.10 (1.00–1.22)*	2,970	1.08 (0.85–1.37)	650	1.10 (0.86–1.41)	605
NO_2_	1.10 (1.01–1.21)*	3,642	1.14 (1.03–1.27)*	2,970	1.10 (0.85–1.42)	650	1.14 (0.87–1.49)	605

ORs are calculated as described in “Materials and Methods.”

a Adjusted for mother smoking during pregnancy, ETS exposure, mother’s/father’s education, sex, gas for cooking/heating, siblings, breast-feeding, molds, pets, parental allergy, mother’s age; in the Netherlands only, adjusted for ethnicity, study arm (intervention/natural history), and use of allergen-impermeable mattress cover.

* Statistically significant elevated ORs (*p* < 0.05).
